# Rapid Isolation and Multiplexed Detection of Exosome Tumor Markers Via Queued Beads Combined with Quantum Dots in a Microarray

**DOI:** 10.1007/s40820-019-0285-x

**Published:** 2019-07-16

**Authors:** Yanan Bai, Yunxing Lu, Kun Wang, Zule Cheng, Youlan Qu, Shihui Qiu, Lin Zhou, Zhenhua Wu, Huiying Liu, Jianlong Zhao, Hongju Mao

**Affiliations:** 1 State Key Laboratory of Transducer Technology, Shanghai Institute of Microsystem and Information Technology, Chinese Academy of Sciences, Shanghai, 200050 People’s Republic of China; 2 Center of Materials Science and Optoelectronics Engineering, University of Chinese Academy of Sciences, Beijing, 100049 People’s Republic of China; 3 School of Stomatology, Dalian Medical University, Dalian, 116044 People’s Republic of China

**Keywords:** Exosome, Liquid biopsy, Quantum dot, Multiplexed detection, Cancer diagnosis

## Abstract

Tumor-derived exosomes are actively involved in cancer progression and metastasis and have emerged as a promising marker for cancer diagnosis in liquid biopsy. Because of their nanoscale size, complex biogenesis, and methodological limitations related to exosome isolation and detection, advancements in their analysis remain slow. Microfluidic technology offers a better analytic approach compared with conventional methods. Here, we developed a bead-based microarray for exosome isolation and multiplexed tumor marker detection. Using this method, exosomes are isolated by binding to antibodies on the bead surface, and tumor markers on the exosomes are detected through quantum dot (QD) probes. The beads are then uniformly trapped and queued among micropillars in the chip. This design benefits fluorescence observation by dispersing the signals into every single bead, thereby avoiding optical interference and enabling more accurate test results. We analyzed exosomes in the cell culture supernatant of lung cancer and endothelial cell lines, and different lung cancer markers labeled with three QD probes were used to conduct multiplexed detection of exosome surface protein markers. Lung cancer-derived samples showed much higher (~ sixfold–tenfold) fluorescence intensity than endothelial cell samples, and different types of lung cancer samples showed distinctive marker expression levels. Additionally, using the chip to detect clinical plasma samples from cancer patients showed good diagnostic power and revealed a well consistency with conventional tests for serological markers. These results provide insight into a promising method for exosome tumor marker detection and early-stage cancer diagnosis.

**Electronic supplementary material:**

The online version of this article (10.1007/s40820-019-0285-x) contains supplementary material, which is available to authorized users.

## Highlights

- A bead-based microarray for exosome isolation and multiplexed tumor marker detection was developed.
- The beads are uniformly trapped and queued among the micropillars in the chip, which can avoid optical interference and enable more accurate test results.
- The results with different types of lung cancer exosome samples showed distinctive marker expression levels.

**Electronic supplementary material:**

The online version of this article (10.1007/s40820-019-0285-x) contains supplementary material, which is available to authorized users.

## Graphical abstract

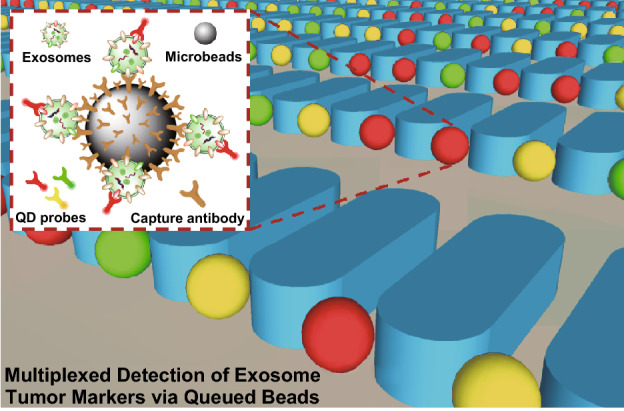

## Introduction

Exosomes are nanosized membrane-bound vesicles (~ 30–150 nm) secreted by most cells through the endolysosomal pathway [1] and package of lipids, proteins, mRNAs, microRNAs [2], and mitochondrial DNA [3] originating from their parent cells [4]. Exosomes play a vital role in intercellular communication [5], especially tumor-derived exosomes, which participate in cancer progression and metastasis actively [6, 7]. Exosomes, together with circulating tumor cells (CTCs) and circulating tumor DNA (ctDNA), are the three major markers for liquid biopsy, which offer a noninvasive solution to early detection, diagnosis, and prognosis of cancer patients [8–10]. Compared with CTCs, ctDNA, and traditional well-studied serological protein markers, exosomes show advantages in their abundance and stability [11, 12]. Approximately one billion exosomes can be detected in one milliliter human blood, and exosomes can maintain an intact biological structure because of their protective phospholipid bilayer [13].

However, studies of exosomes have been severely hampered by difficulties in isolation and detection because of their small size and complex origin [14]. The most widely adopted isolating method is density-gradient ultracentrifugation, but it requires expensive equipment and extensive time [15]. Additionally, ultracentrifugation cannot isolate other vesicles or large protein aggregates similar in size to exosomes, resulting in unnecessary contamination. And traditional detection methods, such as western blot, ELISA, and flow cytometry, need large amounts of purified exosomes from blood, biological fluids, or cell culture supernatant. Therefore, an efficient, reliable, and economical method to isolate and detect exosomes is an urgently target.

With the development of microfluidic technology, many progress in the fields of biology, medicine, and tumor diagnosis has been made [16–18]. In particular, it offers a promising solution to exosome isolation and detection, with advantages including low-volume consumption, high sensitivity, and quick reaction time [19, 20]. Several microfluidic methods for exosome research have been reported [21]. Briefly, isolating exosomes with microfluidic methods can be divided into two types: size-based and immuno-based. Size-based methods physically sort exosomes within a certain diameter range. Wang et al. reported a method using ciliated micropillars to isolate exosomes in biological fluids [22], and Wunsch et al. isolated exosomes from other vesicles using a nanoscale lateral displacement array [23]. Size-based methods sort exosomes with good homogeneity, but cannot exclude similar-sized nanoparticles. Immuno-based methods isolate exosomes through antigen–antibody hybridization with common exosome markers, such as CD9, CD81, and CD63 [4]. Chen et al. developed a microfluidic chip to isolate exosomes using an anti-CD63-coated polydimethylsiloxane (PDMS) surface, which involved complicated chip fabrication [24]. Zeng et al. applied immuno-magnetic bead-based methods for intravesicular protein marker detection in 2014 [25] and surface protein marker detection in 2016 [26], and the latter can be directly applied for plasma sample detection and achieve on-chip analysis; however, both methods require an external magnetic field, and the polymerized immuno-magnetic beads can potentially cause interference during fluorescence observation. Yadav et al. developed an electrochemical method for the detection of disease-specific exosomes using the advantages of screen-printed electrodes and electrochemical readout [27]; Sina et al. presented a simple exosome quantification method using surface plasmon resonance (SPR) platform [28]; however, both methods require pre-isolation of the exosomes using the total exosome isolation reagent (Life Technologies cat no #4478359).

Here, we report the immuno-based microfluidic chip for rapid exosome isolation and multiplexed surface marker detection via queued beads in the microarray. A bead-based sandwich immunoassay was established, where anti-CD9 labeled microbeads capture exosomes and QD probes with tumor-specific antibodies bind to exosomes on the bead surface. We also designed a micropillar array to enable uniform bead distribution, so that the queued beads could benefit subsequent fluorescence analysis. This chip was employed to analyze exosomes from cell culture supernatant and clinical plasma samples, and the whole isolation and detection process finished within 1 h. We observed distinctive levels of fluorescence intensity between lung cancer samples and healthy controls along with robust diagnostic power and consistency with conventional tests. The results indicated that this chip can offer a novel approach for cancer diagnosis via exosome tumor markers and promote the development of liquid biopsy in clinical applications.

## Materials and Method

### Materials

Sulfo-*N*-hydroxysulfosuccinimide (NHS), 1-ethyl-3-[3-dimethylaminopropyl] carbodiimide (EDC), trichloro (1H,1H,2H,2H-perfluorooctyl) silane (PFOTS), Tween-20, and bovine serum albumin (BSA) were purchased from Sigma-Aldrich (St. Louis, MO, USA). PDMS was purchased from Dow Corning (NY, USA). Microbeads were purchased from BaseLine ChromTech (Tianjin, China). QDs were purchased from Jiayuan Quantum Dot (Wuhan, China). Anti-CEA and anti-Cyfra21-1 were purchased from Medix Biochemica (Kauniainen, Finland). Anti-ProGRP was purchased from Linc-Bio Science (Shanghai, China). Anti-CD9 was purchased from Ancell (Bayport, USA). Phosphate-buffered saline (PBS) was purchased from Sangon Biotech (Shanghai, China). Fetal bovine serum (FBS), Roswell Park Memorial Medium (RPMI) 1640, and penicillin–streptomycin were purchased from Gibco Thermo Fisher Scientific (Waltham, MA, USA).

### Preparation of Exosome-Capture Beads and QD Probes

Carboxylic cross-linked polystyrene microbeads were activated through incubation with sulfo-NHS and EDC under acidic conditions (pH 5.0) for 2 h at 25 °C, followed by mixing the activated beads with exosome-capture antibodies (anti-CD9) to bind with amine groups on the antibodies. After incubation at 37 °C for 2 h, the exosome-capture beads were resuspended in 1 × PBS with 1% BSA and stored at 4 °C until use.

QD probes with detection antibodies were prepared based on the coupling of the thiols (–SH) on the antibodies to the maleimide-activated surface of the QDs by the method described previously [29]. QD probes for carcinoembryonic antigen (CEA; excitation: 490 nm, emission: 625 nm), fragments of cytokeratin 19 (Cyfra21-1; excitation: 490 nm, emission: 525 nm), and pro-gastrin-releasing peptide (ProGRP; excitation: 490 nm, emission: 585 nm) were functionalized for subsequent analysis.

### Chip Design and Fabrication

The chip was fabricated according to the standard method for rapid prototyping of microfluidic systems in PDMS [30]. The structure of the micropillar array depicted via CAD software was projected onto a photo-mask and then fabricated through photolithography and deep reactive ion etching techniques onto the silicon wafer. The wafer was silanized via treatment with self-assembled monolayers of PFOTS to form an anti-adhesive layer [31]. A 10:1 (w/w) mixture of PDMS base and curing agent was vacuumized and then poured over the silicon master. After a 2-h incubation at 95 °C, the PDMS was cured and punched at inlet and outlet ports. After air plasma treatment, the PDMS chip was bound firmly to a glass slide (Fig. S1).

### Chip Operation

Samples, exosome-capture beads, and QD probes were mixed together and incubated under vibration at 37 °C for 20 min. The mixture was then introduced into the vacuumized chip by negative pressure through a syringe pump (PHD 2000; Harvard Apparatus, MA, USA). Briefly, a blocking buffer (1 × PBS buffer with 0.1% BSA and 0.05% Tween-20) was first introduced into the chip for 5 min to minimize non-specific adsorption of the PDMS chip. Subsequently, the mixture of samples, beads, and QD probes was introduced into the chip, during which the beads would distribute uniformly along the microarray. Finally, a washing buffer (1 × PBS buffer with 0.05% Tween-20) was introduced into the chip for 5 min to wash out the excessive samples and QD probes. The whole injection process was performed under a flow rate of 3 µL min^−1^.

### Cell Culture and Clinical Sample Collection

The A549, H226, H446 (derived from lung adenocarcinoma, lung squamous carcinoma, small cell lung cancer, respectively), and human umbilical vein endothelial cell (HUVEC) lines were purchased from Cell Bank (Shanghai Institutes for Biological Sciences, Shanghai, China). All cells passed tests for mycoplasma contamination and were cultured in RPMI 1640 supplemented with 10% FBS, 1% (v/v) penicillin–streptomycin in a Forma direct-heat CO_2_ incubator (Thermo Fisher Scientific, Waltham, MA, USA) at 5% CO_2_ and 37 °C.

Plasma samples were obtained from 10 patients who had not undergone primary surgical resection of lung cancer and 10 healthy controls in 2017 at Shanghai Zhongshan Hospital. Healthy controls were recruited from people who underwent a routine health checkup and showed no disease. Demographic and clinical characteristics of subjects are summarized in Table S1. All subjects gave informed consent prior to sample collection. The pathological stage of each sample was determined by an experienced pathologist according to the TNM (Tumor–Node–Metastasis) Classification of Malignant Tumors. All aspects of this study were approved by the Institutional Review Board of Shanghai Zhongshan Hospital, China. Patients with lung cancer with incomplete medical records, prior chemotherapy or radiation, lost to follow-up, or withdrawal of consent were excluded from this study. Peripheral blood samples were collected by venipuncture from all subjects. Cell-free plasma was isolated using a two-step centrifugation protocol, 1900×*g* for 10 min and 3000×*g* for 15 min at 4 °C, followed by further experiment or storage at − 80 °C.

### Model Exosome Isolation by Ultracentrifugation

Model exosome samples were collected from cell culture supernatant through ultracentrifugation. The cell culture medium was replaced with serum-free medium once the cells reached ~ 70–80% density. After 36-h starvation culture, 10 mL of cell culture supernatant was collected for exosome isolation. The isolation process involves a series of centrifugation steps at 4 °C [15]. Briefly, the cell culture supernatant was initially centrifuged at 2000×*g* for 20 min to eliminate the remaining cells. Subsequently, centrifugation at 10,000×*g* for 30 min was conducted to eliminate the cell debris. Finally, exosomes were purified through ultracentrifugation at 100,000×*g* for 70 min twice. After that, the isolated exosomes were resuspended in 150 µL of PBS.

### Characterization of Exosomes

Nanoparticle tracking analysis (NTA) of exosome samples was conducted by the ZetaView Nanoparticle Tracking Analyzer (Particle Metrix, Meerbusch, Germany) following the standard protocols [32]. Exosomes isolated through ultracentrifugation were diluted in PBS (1:100) and loaded into the flushed chamber. Analyses were automatically carried out, and the concentration and diameter of exosomes were then measured and analyzed through the corresponding software ZetaView 8.03.04.01.

Exosomes bonded on the surface of microbeads were characterized by scanning electron microscope (SEM) [33]. Approximately 1000 beads were mixed with 1 mL of cell culture supernatant. After incubation at 37 °C for 20 min, the mixture was centrifuged at 1000×*g* for 10 min and resuspended in 100 µL of PBS. The samples were fixed in 4% paraformaldehyde for 1 h, dehydrated in a series of increasing ethanol concentrations, uniformly spread onto a silica glass, and then lyophilized overnight. After that, the silica glass was vacuumized and sputter-coated with gold at room temperature for 60 s. Finally, the morphology of exosomes on microbeads was examined under a field emission scanning electron microscope (JSM-7800F; JEOL, Tokyo, Japan).

### Data Collection and Analysis

Images were obtained using a CCD camera (DP80; Olympus, Tokyo, Japan) on an inverted fluorescence microscope (IX51; Olympus, Tokyo, Japan). Bright-field images were acquired using a 20 × lens at a 160 ms exposure time. Fluorescence images were acquired using a 40 × lens through a filter (~ 460–495 nm) at a 3.25 s exposure time and an 1800 black-balance value. QD probes for CEA, Cyfra21-1, and ProGRP were exposed to light from 460 to 495 nm wavelength to emit red, green, and yellow fluorescence, respectively. Fluorescence intensity was calculated using Image Pro Plus 6.0 software (Mediacy Cybernetics, Inc., Rockville, MD, USA), and the average optical density of each bead was acquired.

## Results and Discussion

### Bead-Based Exosome Microfluidic Chip

**Fig. 1:**
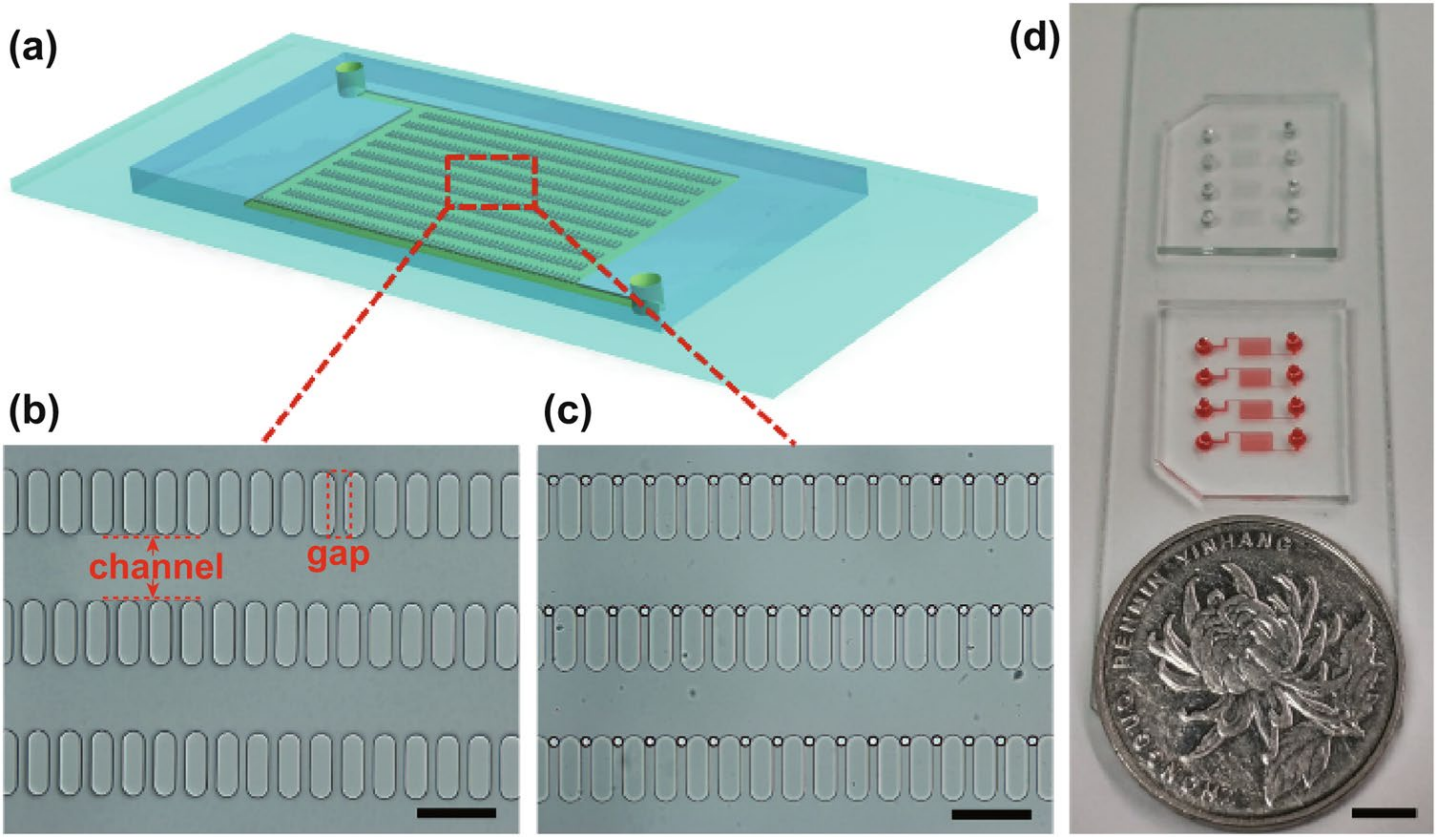
Bead-based exosome microfluidic chip. **a** Schematic diagram of the PDMS chip containing a microarray for uniform distribution of microbeads. **b** Microarray structure in the chip and **c** microbeads queued in lines along the microarray. Scale bar, 100 µm. **d** Photograph of the prototype microfluidic chip. Scale bar, 50 mm

To distribute microbeads (15 µm) in the chip uniformly, we designed a microfluidic chip (Fig. 1a) comprising a PDMS slab bonded with a glass slide. The main structure in the PDMS was a microarray containing 604 micropillars, with each pillar 90 µm long, 30 µm wide, 18 µm high, and the inlet and outlet set at the diagonal of the chip. Figure 1b shows a microscopic image of the chip, where the channel between the adjacent micropillar lines was 90 µm wide, and the gap between adjacent pillars was 14 µm wide. After flushing with the blocking buffer, beads were introduced into the chip, trapped at the gaps, and queued in lines along the microarray (Fig. 1c). Figure 1d shows the device with two PDMS chips bonded onto one glass slide and each chip consisting of four microarray structures. The real PDMS chip was ~ 150 mm wide.

### Working Principle of the Chip

**Fig. 2:**
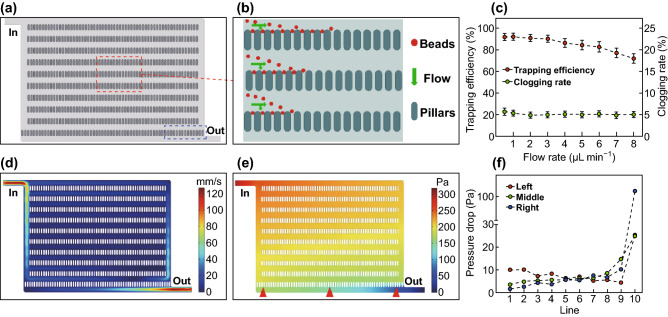
Working principle and simulation of the chip. **a** Two-dimensional structure of the microarray. The control district is labeled by the blue dashed box. **b** Schematic diagram of beads trapped at gaps along the microarray. **c** Influence of flow rate on trapping efficiency and clogging rate. **d** Flow velocity profile and **e** pressure profile of the fluid dynamics in the chip using a velocity of 88 mm s^−1^ at the inlet. **f** The variation of pressure drop at the left, middle, and right gaps from lines one to ten according to point evaluation. (Color figure online)

The two-dimensional structure of the microarray is shown in Fig. 2a. The microarray comprised 10 lines of micropillars, with two additional micropillars placed on both sides of the last line in order to intercept the missing beads, which would aggregate at the control district close to the outlet labeled by the blue dashed box. This would prevent beads from escaping the chip and avoid data loss. As shown in Fig. 2b, beads flowing in the channels were deflected by the pressure difference in the adjacent channels and trapped at the gaps. Once a gap was blocked by a bead, the flow resistance of that gap would increase dramatically, and subsequent beads would bypass the site and flow to other empty gaps. In this case, beads would not aggregate at the same gap, thereby allowing uniform distribution.

We observed that the flow rate significantly influenced bead interception. Therefore, a flow rate gradient ranging from 0.5 to 8 µL min^−1^ was established to investigate the optimal flow rate for bead interception. Two parameters were considered: trapping efficiency and clogging rate. Trapping efficiency was defined as the proportion of the number of beads trapped at the gaps, except that of the control district, to the total number of beads injected into the chip. The clogging rate was defined as the proportion of gaps trapping plural beads relative to the total number of gaps, except that of the control district. As shown in Fig. 2c, the trapping efficiency remained relatively steady (~ 90%) before the flow rate reached 3 µL min^−1^ and decreased rapidly once the flow rate exceeded 3 µL min^−1^. The clogging rate fluctuated slightly (~ 5%) along with the increasing flow rate; therefore, we speculated that the clogging rate had no necessary connection with the flow rate at higher flow rates. According to our observation, the inhomogeneity of the beads and the PDMS fragment generated during the punch were more likely to cause bead clogging. Therefore, 3 µL min^−1^ was adopted as the optimal flow rate.

To clarify the mechanism associated with this uniform distribution phenomenon, fluid dynamics in the chip was analyzed by performing velocity and pressure simulation through Multiphysics 5.2 software (COMSOL, Inc., Burlington, MA, USA) [34, 35]. The simulation was based on the steady-state Navier–Stokes’ equation for incompressible fluid, and two-dimensional laminar flow was studied. The model geometry was the same size as the actual chip structure, and the flow velocity at the inlet was set as 88 mm s^−1^ according to the calculation using a high-speed camera under an injection rate of 3 µL min^−1^ (Fig. S2). Through the simulation, the flow velocity profile (Fig. 2d) and the pressure profile (Fig. 2e) were acquired. The micropillar lines from inlet to outlet were defined as lines 1 to 10, and the pressure drop at the left, middle, and right (the red triangle in Fig. 2e) gaps of each line (except the additional micropillars) was calculated through point evaluation. Figure 2f shows the pressure drop of the target gaps from lines 1 to 10. We found that the pressure drop from lines 1 to 9 of the left gaps gradually decreased, whereas the pressure drop at the middle and right lines increased gradually, but generally remained at a relatively narrow range (~ 10 Pa) as compared with the dramatic increase in pressure drop at the last line. The steady state of the pressure drops from lines 1 to 9 ensured a stable interception of beads, and the dramatic increase in pressure drop at the last line might have provided a driving force for the beads flowing to the outlet.

### Characterization of the Bead-Based Exosome Immunoassay

**Fig. 3:**
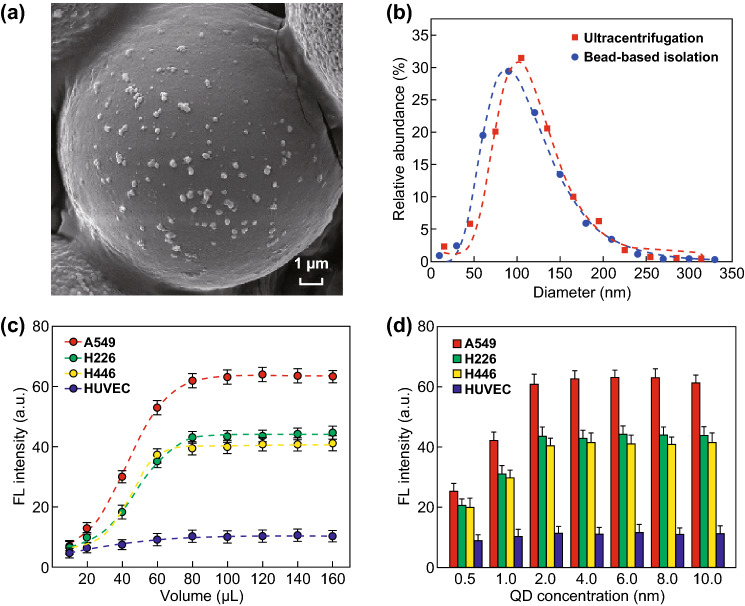
Morphological characterization of exosomes and optimization of the immunoassay system. **a** SEM image of exosomes bound to the bead surface. **b** Diameter distribution of bead-based isolated exosomes using particle analysis of SEM images (*R*^2^ = 0.996) compared with NTA of ultracentrifugation-isolated exosomes (*R*^2^ = 0.982). Dashed lines represent log-normal fitting. **c**, **d** Variation in the fluorescence intensities of different cell lines according to increasing volumes of cell culture supernatant (**c**) and QD concentrations (**d**). Dashed lines represent sigmoidal dose–response fitting (*R*^2^ > 0.99)

We used anti-CD9 labeled microbeads to isolate exosomes from cell culture supernatant. Morphological characterization of exosomes captured on the beads was conducted by SEM (Fig. 3a). To verify the results of bead-based isolation, we conducted NTA of exosomes isolated by ultracentrifugation from the same source in order to measure their diameter distribution. The diameters of exosomes on the bead surface were calculated using particle analysis of the SEM images. Exosomes isolated using our methods exhibited a good consistency in diameter distribution with those isolated by ultracentrifugation (Fig. 3b), suggesting our bead-based method offered reliable specificity compared with conventional methods.

Exosome tumor markers were detected by sandwich immunoassay through microbeads, exosomes, and QD probes (Fig. S3). All microbeads needed to be saturated with exosomes (Fig. S4), ensuring the consistent detection of different samples. To determine the adequate sample volume of cell culture supernatant, we evaluated supernatant volumes from 10 to 160 µL with ~ 500 microbeads (Fig. S5) and excess amounts of CEA-QD probes. Figure 3c shows the relationship between fluorescence intensity and sample volume fitted according to sigmoidal dose–response characteristics (*R*^2^ > 0.99). The fluorescence intensity of A549, H226, and H446 cell culture supernatants initially increased rapidly, smoothed gradually along with increased volumes, and remained relatively steady at 80 µL. The fluorescence intensity associated with HUVEC culture supernatant increased slightly, and we speculated that this was due to exosomes secreted by non-cancer cells carrying few surface CEA markers. Based on these results, 80 µL was adopted as the optimal supernatant volume. Because excessive amounts of QD probes might cause unnecessary optical interference, we further optimized the amount of QD probes used for detection. Figure 3d shows the enhanced fluorescence intensity obtained using increasing concentrations of QD probes, with an optimal concentration of 2 nM ultimately determined. These data indicated that ~ 500 beads, 80 µL cell culture supernatant, and 2 nM QD probes represented the optimal experimental protocol for this immunoassay system.

### Multiplexed Detection of Exosome Tumor Markers from Cell Culture Supernatant

Single marker detection for lung cancer was limited in sensitivity and specificity; therefore, we conducted multiplexed detection by targeting three lung cancer-related markers: CEA, a broad-spectrum tumor protein marker most commonly observed in adenocarcinoma [36]; Cyfra21-1, allowing measurement of soluble cytokeratin-19 fragments in serum and a useful marker for squamous cancer [37]; and ProGRP, a specific and actively secreted product of small cell lung carcinoma cells [38]. Exosomes from A549, H226, H446, and HUVEC culture supernatant were isolated using anti-CD9-labeled beads, and three QD probes, respectively, labeled with corresponding tumor marker antibodies were used to detect the isolated exosomes on the bead surface. The volume of each component referred to the former part.

**Fig. 4:**
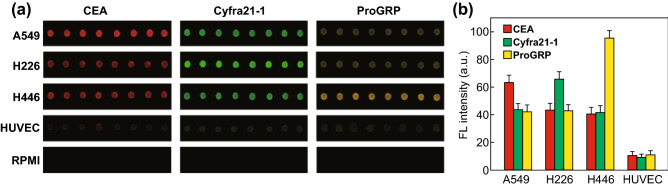
**a** Fluorescence images of exosomes derived from different cell lines using three different tumor-specific QD probes. **b** Average expression levels of three lung cancer markers measured in different cell lines

We observed a macroscopic difference in fluorescence intensity between cancer cells and endothelial cells (Fig. 4a), with RPMI 1640 used as the negative control. The average expression levels of the three lung cancer markers were measured and are shown in Fig. 4b. Lung cancer cells showed higher (~ 6–10-fold) fluorescence intensity as compared with that of endothelial cells, and interestingly, different lung cancer cells showed distinctively different marker expression levels, potentially allowing for further tumor classification.

### Multiplexed Detection of Exosome Tumor Markers from Plasma Samples

**Fig. 5:**
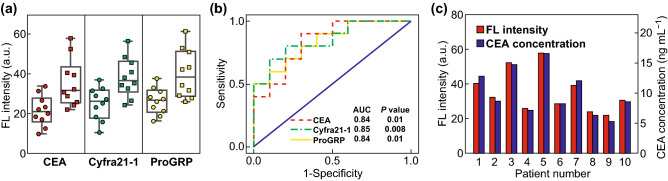
**a** Box–whisker plots with scattering points indicating the expression levels of the three tumor markers from clinical plasma-derived exosomes, lung cancer patients (squares), and healthy controls (circles). **b** ROC curves of the multiplexed detection of three lung cancer exosome markers (CEA: AUC = 0.84, *p* = 0.01; Cyfra21-1: AUC = 0.85, *p* = 0.008; and ProGRP: AUC = 0.84, *p* = 0.01). The confidence interval was 95%. **c** Comparison of clinical data of blood CEA concentrations with exosome CEA expression levels detected using our method

We further employed the chip for plasma-based diagnosis of lung cancer by multiplexed detection of the three tumor markers, with samples from 20 human subjects (10 lung cancer patients and 10 healthy controls) used for detection (Table S1). As the exosome concentration in cell culture supernatant is ~ 10^7^ particles mL^−1^ according to the NTA results, while the exosome concentration in human blood is ~ 10^9^ mL^−1^ [13], 10 µL plasma samples were sufficient for our immunoassay system and the concentration of QD probes were determined as 8 nM. Box–whisker plots with scattering points were derived according to the fluorescence intensities associated with the respective marker expression levels (Fig. 5a). Lung cancer patients showed increased marker expression levels as compared with healthy controls; however, we did not observe distinct marker expression levels associated with different types of lung cancer, which might be attributable to the complex components included in plasma samples. Receiver operator characteristic (ROC) analysis was conducted to evaluate diagnostic accuracy (Fig. 5b), and the area under the ROC curve (AUC) was obtained to evaluate the overall accuracy of the test. Additionally, true positives (sensitivity) and false positives (one specificity) were analyzed to determine diagnostic power. The AUCs obtained for CEA, Cyfra21-1, and ProGRP were 0.84, 0.85, and 0.84 (Fig. S6), respectively, indicating high accuracy in discriminating plasma exosomes from lung cancer patients versus healthy individuals (Table S3).

To assess the reliability of the chip results, we compared the CEA expression levels of 10 lung cancer patients using CEA-QD probes with clinical CEA concentrations obtained from blood test using electro-chemiluminescence immunoassay. A minimal difference was observed between the experiment results and clinical data (Fig. 5c), and Pearson correlation coefficient (*r* = 0.9872) and two-tailed comparison (*p* < 10^−4^) of the results confirmed statistical significance, suggesting the reliability of our detection method.

## Conclusions

In this study, we described a novel microfluidic immunoassay system for exosome isolation and detection using beads queued uniformly at gaps among the microarray. Compared with existing microfluidic methods, our chip exhibited the following advantages: (1) This design was capable of intercepting microbeads at gaps between adjacent micropillars without clogging, thereby avoiding interference from dissociative fluorescence signals; (2) the use of QDs for immunoassay detection improved fluorescence stability and multiplexed biomarker measurement; (3) the simple structure, rapid isolation and detection, and no requirement of external fields makes the chip promising for clinical application. Additionally, the total time necessary for reaction, injection, and observation was less than 1 h, with all reaction components capable of being mixed simultaneously, thereby reducing tedious manual operations.

There were still some limitations in our study. We noticed distinctions between the expression levels of tumor markers in exosomes from different lung cancer cell lines, but failed to obtain similar results in clinical plasma samples. Although we speculated that this might be due to the complexity of plasma composition, the statistical limitation because of the small sample size represents a non-negligible factor. In future work, we will use larger sample sizes to assess the potential of the chip for lung cancer classification and further validate its diagnostic power. These results provide critical insight into the efficacy of this method for future application in clinical testing related to cancer, as well as other diseases.

## Electronic supplementary material

Below is the link to the electronic supplementary material.

Supplementary material 1 (DOCX 5156 kb)

Supplementary material 2 (AVI 5274 kb)

## References

[1] Raposo G, Stoorvogel W (2013). Extracellular vesicles: exosomes, microvesicles, and friends. J. Cell Biol..

[2] Mathivanan S, Fahner CJ, Reid GE, Simpson RJ (2012). ExoCarta 2012: database of exosomal proteins, RNA and lipids. Nucleic Acids Res..

[3] Guescini M, Genedani S, Stocchi V, Agnati LF (2010). Astrocytes and Glioblastoma cells release exosomes carrying mtDNA. J. Neural Transm..

[4] Thery C, Zitvogel L, Amigorena S (2002). Exosomes: composition, biogenesis and function. Nat. Rev. Immunol..

[5] Vlassov AV, Magdaleno S, Setterquist R, Conrad R (2012). Exosomes: current knowledge of their composition, biological functions, and diagnostic and therapeutic potentials. Biochim. Biophys. Acta.

[6] Kalluri R (2016). The biology and function of exosomes in cancer. J. Clin. Invest..

[7] Suchorska WM, Lach MS (2016). The role of exosomes in tumor progression and metastasis (review). Oncol. Rep..

[8] Sharma A, Khatun Z, Shiras A (2016). Tumor exosomes: cellular postmen of cancer diagnosis and personalized therapy. Nanomedicine.

[9] Shao Y, Shen Y, Chen T, Xu F, Chen X, Zheng S (2016). The functions and clinical applications of tumor-derived exosomes. Oncotarget.

[10] Whiteside TL (2015). The potential of tumor-derived exosomes for noninvasive cancer monitoring. Expert Rev. Mol. Diagn..

[11] He M, Zeng Y (2016). Microfluidic exosome analysis toward liquid biopsy for cancer. J. Lab. Autom..

[12] Contreras-Naranjo JC, Wu HJ, Ugaz VM (2017). Microfluidics for exosome isolation and analysis: enabling liquid biopsy for personalized medicine. Lab Chip.

[13] Arraud N, Linares R, Tan S, Gounou C, Pasquet JM, Mornet S, Brisson AR (2014). Extracellular vesicles from blood plasma: determination of their morphology, size, phenotype and concentration. J. Thromb. Haemost..

[14] Colombo M, Raposo G, Thery C (2014). Biogenesis, secretion, and intercellular interactions of exosomes and other extracellular vesicles. Annu. Rev. Cell Dev. Biol..

[15] C. Thery, S. Amigorena, G. Raposo, A. Clayton, Isolation and characterization of exosomes from cell culture supernatants and biological fluids. Curr. Protoc. Cell Biol. Chapter 3, Unit 3.22 (2006). 10.1002/0471143030.cb0322s30 10.1002/0471143030.cb0322s30 18228490

[16] Chen JD, Chen D, Xie Y, Yuan T, Chen X (2013). Progress of microfluidics for biology and medicine. Nano-Micro Lett..

[17] Shikha S, Zheng X, Zhang Y (2018). Upconversion nanoparticles-encoded hydrogel microbeads-based multiplexed protein detection. Nano-Micro Lett..

[18] Li R, Zhou M, Li J, Wang Z, Zhang W (2018). Identifying EGFR-expressed cells and detecting EGFR multi-mutations at single-cell level by microfluidic chip. Nano-Micro Lett..

[19] Shang L, Cheng Y, Zhao Y (2017). Emerging droplet microfluidics. Chem. Rev..

[20] Hu B, Li J, Mou L, Liu Y, Deng J (2017). An automated and portable microfluidic chemiluminescence immunoassay for quantitative detection of biomarkers. Lab Chip.

[21] Boriachek K, Islam MN, Moller A, Salomon C, Nguyen NT (2018). Biological functions and current advances in isolation and detection strategies for exosome nanovesicles. Small.

[22] Wang Z, Wu HJ, Fine D, Schmulen J, Hu Y (2013). Ciliated micropillars for the microfluidic-based isolation of nanoscale lipid vesicles. Lab Chip.

[23] Wunsch BH, Smith JT, Gifford SM, Wang C, Brink M (2016). Nanoscale lateral displacement arrays for the separation of exosomes and colloids down to 20 nm. Nat. Nanotechnol..

[24] Chen C, Skog J, Hsu CH, Lessard RT, Balaj L (2010). Microfluidic isolation and transcriptome analysis of serum microvesicles. Lab Chip.

[25] He M, Crow J, Roth M, Zeng Y, Godwin AK (2014). Integrated immunoisolation and protein analysis of circulating exosomes using microfluidic technology. Lab Chip.

[26] Zhao Z, Yang Y, Zeng Y, He M (2016). A microfluidic ExoSearch chip for multiplexed exosome detection towards blood-based ovarian cancer diagnosis. Lab Chip.

[27] Yadav S, Boriachek K, Islam MN, Lobb R, Möller A (2017). An electrochemical method for the detection of disease-specific exosomes. ChemElectroChem.

[28] Sina AA, Vaidyanathan R, Dey S, Carrascosa LG, Shiddiky MJ, Trau M (2016). Real time and label free profiling of clinically relevant exosomes. Sci. Rep..

[29] Liu L, Wu S, Jing F, Zhou H, Jia C (2016). Bead-based microarray immunoassay for lung cancer biomarkers using quantum dots as labels. Biosens. Bioelectron..

[30] Duffy DC, McDonald JC, Schueller OJ, Whitesides GM (1998). Rapid prototyping of microfluidic systems in poly(dimethylsiloxane). Anal. Chem..

[31] Fan X, Jia C, Yang J, Li G, Mao H, Jin Q, Zhao J (2015). A microfluidic chip integrated with a high-density PDMS-based microfiltration membrane for rapid isolation and detection of circulating tumor cells. Biosens. Bioelectron..

[32] Mehdiani A, Maier A, Pinto A, Barth M, Akhyari P, Lichtenberg A (2015). An innovative method for exosome quantification and size measurement. J. Vis. Exp..

[33] Wan Y, Cheng G, Liu X, Hao SJ, Nisic M (2017). Rapid magnetic isolation of extracellular vesicles via lipid-based nanoprobes. Nat. Biomed. Eng..

[34] Wang K, Zhou L, Wang Z, Cheng Z, Dong H (2018). Uniform distribution of microspheres based on pressure difference for carcinoma-embryonic antigen detection. Sens. Actuators B.

[35] Jin D, Deng B, Li JX, Cai W, Tu L (2015). A microfluidic device enabling high-efficiency single cell trapping. Biomicrofluidics.

[36] Grunnet M, Sorensen JB (2012). Carcinoembryonic antigen (CEA) as tumor marker in lung cancer. Lung Cancer.

[37] Barak V, Goike H, Panaretakis KW, Einarsson R (2004). Clinical utility of cytokeratins as tumor markers. Clin. Biochem..

[38] Molina R, Filella X, Auge JM (2004). ProGRP: a new biomarker for small cell lung cancer. Clin. Biochem..

