# Supplementary material for: Rapid Isolation and Multiplexed Detection of Exosome Tumor Markers Via Queued Beads Combined with Quantum Dots in a Microarray
Source: Nanomicro Lett. 2019 Jul 16;11:59. doi: 10.1007/s40820-019-0285-x (PMC7770845; doi:10.1007/s40820-019-0285-x)
Supplement: Supplementary file 1 — Supplementary material 1 (DOCX 5156 kb) [file 40820_2019_285_MOESM1_ESM.docx]

Supplementary Material

**Rapid Isolation and Multiplexed Detection of Exosome Tumor Markers via Queued Beads Combined with Quantum Dots in a Microarray**

Yanan Bai ^1,2^, Yunxing Lu ^1,2^, Kun Wang ^1,2^, Zule Cheng ^1,2^, Youlan Qu ^1,3^, shihui Qiu ^1^, Lin Zhou ^1^, Zhenhua Wu ^1^, Huiying Liu^3,*^, Jianlong Zhao^1,*^, Hongju Mao ^1,2,*^

^1^ State Key Laboratory of Transducer Technology, Shanghai Institute of Microsystem and Information Technology, Chinese Academy of Sciences, Shanghai 200050, China

^2^ Center of Materials Science and Optoelectronics Engineering, University of Chinese Academy of Sciences, Beijing 100049, China

^3^ School of Stomatology, Dalian Medical University, Dalian 116044, China

Schematic of the chip fabrication process


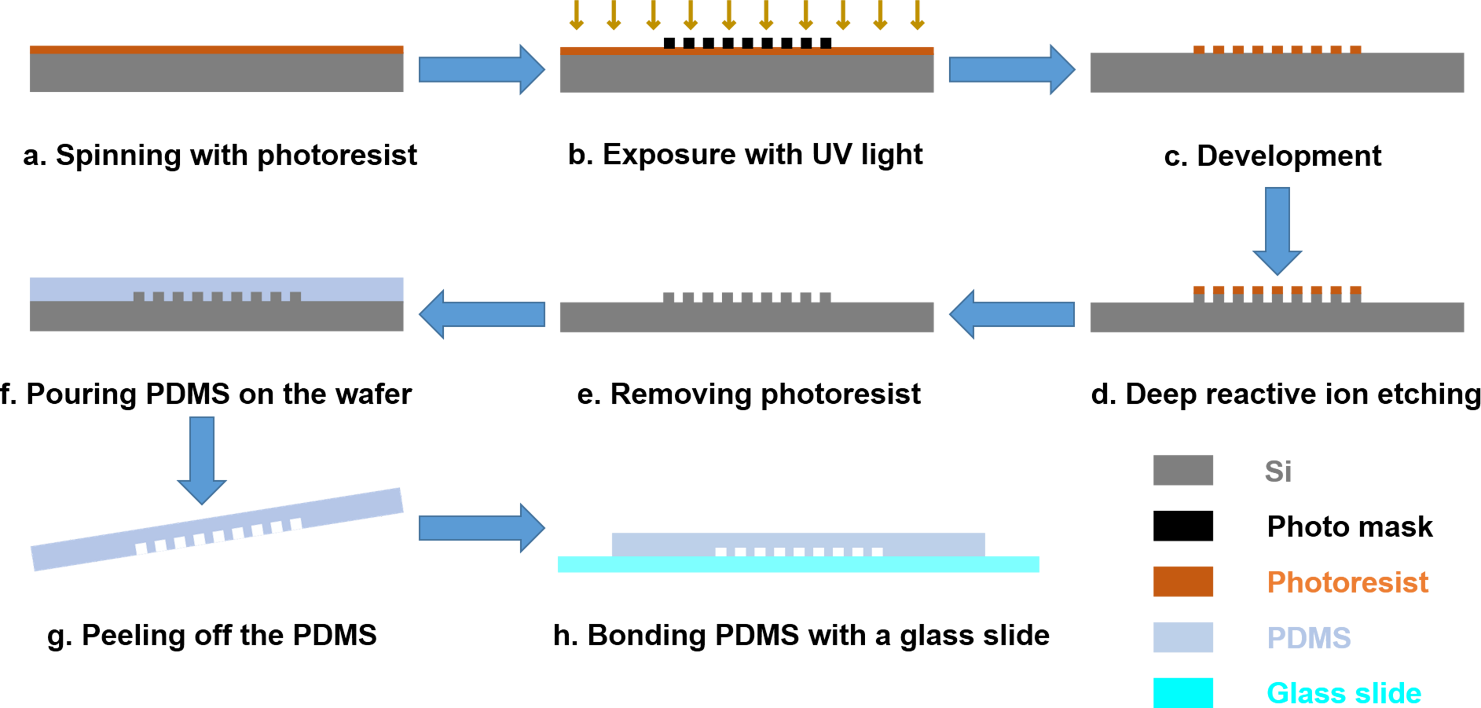


**Fig. S1** Schematic illustration of the total chip fabrication process.

Motion trail of the microbeads under a high speed camera

To figure out the flow velocity of the microbeads in the chip, their movements at the inlet were tracked using the high speed camera on an inverted fluorescence microscope (IX73; Olympus, Tokyo, Japan) under an injection rate of 3 μL/min. Images were acquired using a 10× lens at a 0.1-ms exposure time. A total of 700 images were obtained within 1 s.


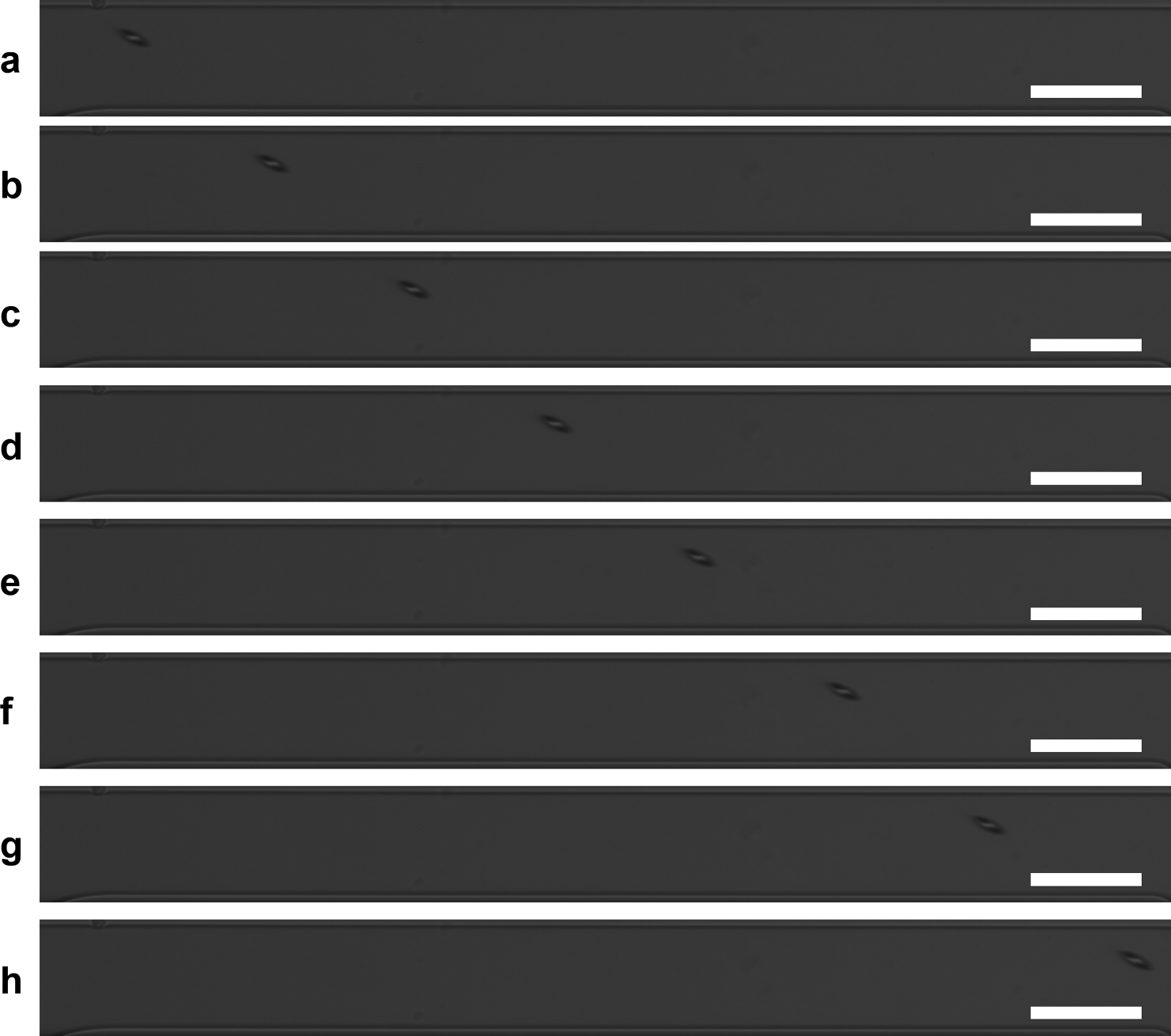


**Fig. S2** The sequential snapshots showing the motion trail of the microbeads under an injection rate of 3 μL/min. 700 images were captured within 1 s. Scale bar, 100 μm.

Sandwich immunoassay of microbeads, exosomes, and QD probes

The testing samples were trisected into three parts and then mixed with microbeads and one specific QD probe respectively. The sandwich immunoassay was conducted through anti-CD9 labelled microbeads capturing exosomes and QD probes with tumor-specific antibodies binding to exosomes on the bead surface.


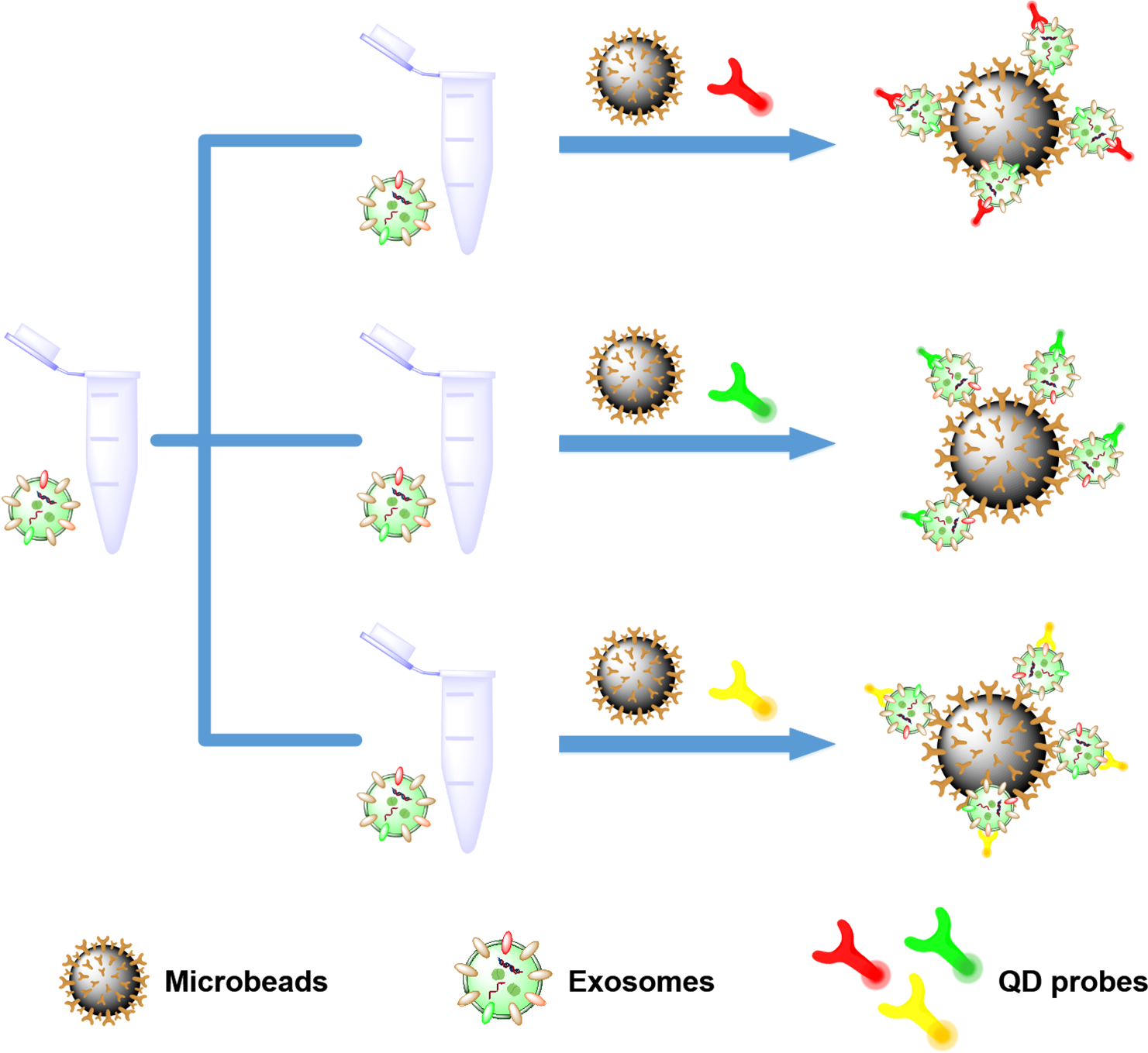


**Fig. S3** Schematic illustration of the sandwich immunoassay of microbeads, exosomes, and QD probes.

Quantification of exosomes on microbeads

To ensure that every microbead is saturated with exosomes, excessive amount of cell culture supernatant (1 mL) from each cell line was mixed with ~1000 beads. Fig. S4 (a) ~ (d) shows the SEM images of exosomes from each cell line captured on the bead surface, with particle analysis of SEM images showing that a single microbead captures almost the same amount of exosomes from different cell lines (Fig. S4 (e)).


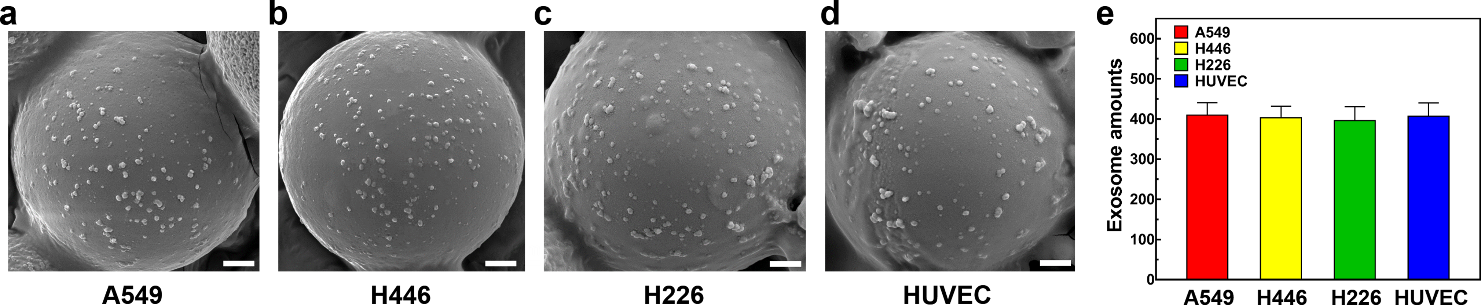


**Fig. S4** (a) ~ (d) SEM images of exosomes on microbeads from each cell line. Scale bar, 1 μm. (e) Average exosome amount on a single bead through particle analysis of SEM images.

Optimization of the bead amount

To figure out the adequate amount of microbeads injected into the chip, we evaluated microbeads with an amount from 100 to 1000 under a flow rate of 3 μL/min. As shown in Fig. S5, the trapping efficiency decreased rapidly once the bead amounts exceeded 500 and the filling rate was nearly saturated after injecting over 600 microbeads. To ensure the robustness of our detection, the amount of beads was finally decided at 500. Trapping efficiency and filling rate was considered: Trapping efficiency was defined as the proportion of the number of beads trapped at the gaps, except that of the control district, to the total number of beads injected into the chip. Filling rate was defined as the gaps that trap a single bead or plural beads relative to the total number of gaps.


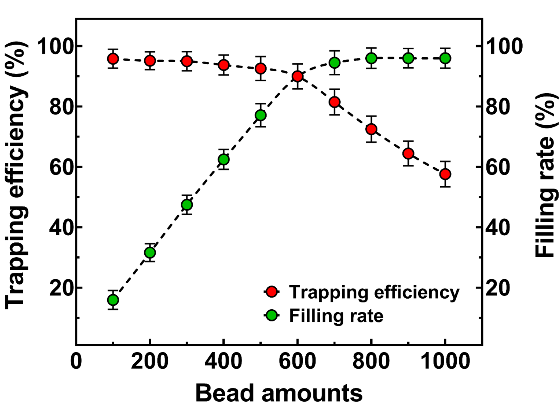


**Fig. S5** The relationship of bead amounts with trapping efficiency and filling rate.

**Table S1.** Clinical pathological characteristics of 10 patients with lung cancer and 10 healthy controls.

| Characteristics | Patients  (n = 10) | Healthy controls  (n = 10) |
| --- | --- | --- |
| Age  Mean ± SD | 59.1 ± 12.2 years | 44.7 ± 5.8 years |
| Gender  Male  Female | 8  2 | 5  5 |
| TNM stage  IIIa  IV | 5  5 |  |
| Type  lung adenocarcinoma (ADC)  lung squamous cell carcinoma (LSCC)  small cell lung cancer (SCLC) | 4  4  2 |  |

Diagnostic Accuracy^1^

Specificity and sensitivity are terms used to assess a clinical test. Receiver operator characteristic (ROC) curve is a plot of (1−specificity) of a test on the x-axis against its sensitivity on the y-axis for all possible cut-off points. The area under this curve (AUC) represents the overall accuracy of a test, with a value approaching 1.0 indicating a high sensitivity and specificity. The AUC is a global measure of diagnostic accuracy. The accuracy classification for a diagnostic test is listed below.

**Table S2** Accuracy classification by AUC for a clinical diagnostic test.

| AUC Range | Classification |
| --- | --- |
| 0.9~1.0 | Excellent |
| 0.8~0.9 | Good |
| 0.7~0.8 | Worthless |
| 0.6~0.7 | Not good |

We analyzed the specificity and sensitivity using ROC curves for on-chip measurements (expression levels of CEA, Cyfra21-1, and ProGRP) from 20 human subjects (10 lung cancer patients, 10 healthy individuals). On-chip assay of multiple exosome tumor markers showed good diagnostic accuracy (CEA: AUC=0.84; Cyfra21-1: AUC=0.85; ProGRP: AUC=0.84).


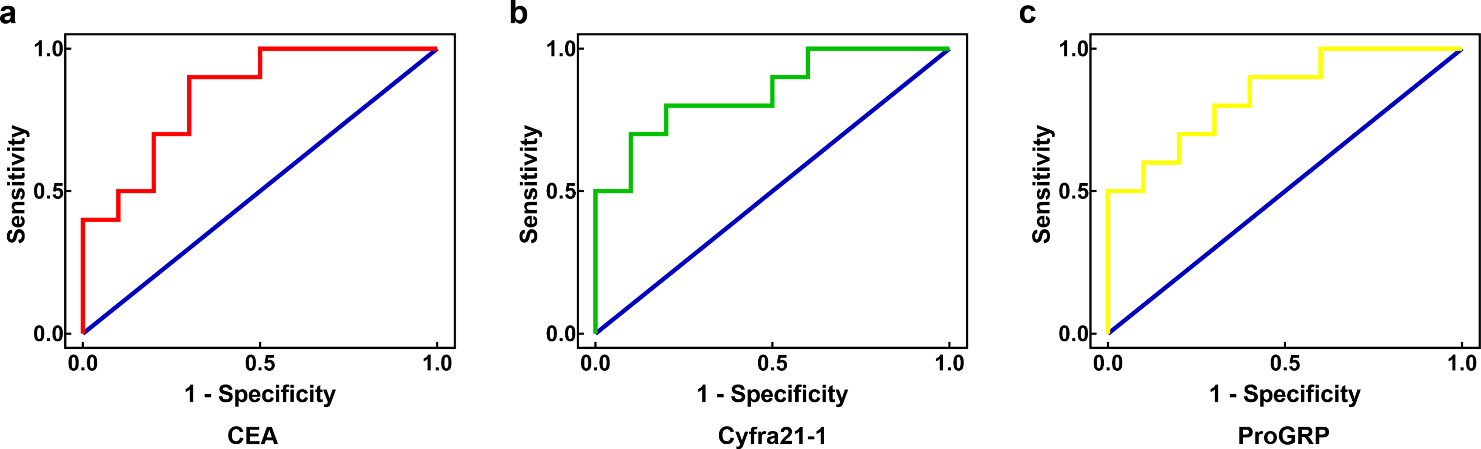


**Fig. S6** ROC curves of the multiplexed detection of three lung cancer exosome markers (CEA, Cyfra21-1, and ProGRP).

**Table S3** Diagnostic accuracy analysis using the receiver operating characteristic curve.

| **Test variables** | **CEA** | **Cyfra21-1** | **ProGRP** |
| --- | --- | --- | --- |
| Area | 0.84 | 0.85 | 0.84 |
| Std.Error | 0.8886 | 0.08652 | 0.08784 |
| 95% confidence interval | 0.6658 to 1.014 | 0.6804 to 1.02 | 0.6678 to 1.012 |
| P value | 0.010165 | 0.0082 | 0.0102 |

Supplementary video

All videos were generated from sequential images captured at 12.5 frames per second under a 10 lens with an inverted fluorescence microscope (IX51; Olympus, Tokyo, Japan) connected to a CCD camera (DP80; Olympus, Tokyo, Japan). The injection is under a flow rate of 3 μL/min.

Supplementary video Sequential images showing the bead-trapping process, where microbeads (15 μm) were deflected into vacant gaps and bypassed bead-blocking gaps.

References

1. Zou, K.H., O'Malley, A.J. & Mauri, L. Receiver-operating characteristic analysis for evaluating diagnostic tests and predictive models. Circulation 115, 654-657 (2007).
